# Engineering WO_3_ Nanostructures via Carboxylic Acid Anodization for Advanced Lithium-Ion Battery Anodes

**DOI:** 10.3390/ma18245602

**Published:** 2025-12-13

**Authors:** Elianny Da Silva, Javier Estarelles Nácher, Rut Sanchis, Vicenta González, Gemma Roselló-Márquez, Ramon Manuel Fernández-Domene, Rita Sánchez-Tovar, Benjamin Solsona

**Affiliations:** Department of Chemical Engineering (ETSE), Universitat de València, Av. Universitat s/n, 46100 Burjassot-Valencia, Spain; elianny.silva@uv.es (E.D.S.); esnaja@alumni.uv.es (J.E.N.); rut.sanchis@uv.es (R.S.); vicenta.gonzalez@uv.es (V.G.); gemma.rosello@uv.es (G.R.-M.); ramon.fernandez@uv.es (R.M.F.-D.)

**Keywords:** WO_3_ nanostructures, carboxylic acids, electrochemistry, Li-ion batteries

## Abstract

WO_3_ nanorods were fabricated following electrochemical anodization of tungsten, under controlled hydrodynamic conditions, in electrolytes containing three distinct carboxylic acids: citric, tartaric and L-aspartic acids, to study the influence of these complexing agents on the morphology and arrangement of the oxide layers. The samples were characterized by FESEM, TEM and XRD, and electrochemical analyses (EIS and ECSA) to assess their potential as anode materials for lithium-ion batteries. This characterization showed the nanostructures anodized in the presence of tartaric acid exhibit uniform morphology and lower total charge transfer resistance associated with the nanostructured layer of WO_3_ and cycling stability, resulting in more efficient electrochemical processes, better conductivity and stability, making these nanostructures promising for anodes in lithium-ion batteries. The cycling of the batteries was also conducted to understand the behavior of the nanostructures as anodes against metallic lithium. The results showed that the nanostructures analyzed in the presence of tartaric acid exhibited the best initial specific capacity, improving the capacity provided by the graphite ones. These samples also showed a good recovery after faster cycling. These findings demonstrate the effectiveness of complexing-agent-assisted anodization as a strategy for tailoring WO_3_ nanostructures with enhanced electrochemical performance.

## 1. Introduction

Currently, most of the global energy consumption is still met by fossil fuels (~82%) [1]. The continuous growth in global energy demand, which has accelerated since the Industrial Revolution, has led to a significant increase in greenhouse gas (GHG) emissions, mainly carbon dioxide (CO_2_), into the atmosphere. It is broadly recognized that the rise in GHG concentrations is the primary driver of major environmental issues such as global warming and the ensuing climate change [2].

The ongoing energy crisis represents a challenge that must be addressed in the near future, promoting a transition toward a sustainable and low-carbon energy model. This transition not only requires the adoption of cleaner and less fossil-dependent energy sources but also improvements in energy utilization and storage efficiency to balance production capacity and consumption demand. However, the intermittent nature of many renewable energy sources hinders their effective integration into the global energy system [1,2]. Therefore, the development of advanced energy storage technologies is essential to ensure supply stability and continuity, allowing renewable energies, clean and fossil-free, to dominate the energy production landscape [1,2].

In this context, nanotechnology has emerged as a promising field for the development of advanced energy storage systems [3,4,5,6,7,8,9,10]. The growing usage of compact electronic devices and the widespread adoption of electric vehicles further emphasize the need for cleaner, more efficient, and higher-capacity energy storage solutions. Among these technologies, electrochemical batteries play a pivotal role. Nevertheless, electrochemical batteries face several challenges, including limited reaction kinetics, stability in the long-term, and safety concerns. It is therefore crucial to foster innovation in the design of novel electrode materials capable of enhancing battery performance and lifespan. Lithium-ion batteries (LIBs) stand out due to their high energy density, long cycle life, low toxicity, and lack of memory effect [11,12,13]. Because of these properties, LIBs outperform supercapacitors in energy storage capacity [14,15,16].

An ideal anode material for LIBs should consist of elements with low atomic weight and density, accommodate large amounts of lithium per formula unit, exhibit long-term cyclability and stability, and possess a high specific capacity with minimal irreversible loss. Graphite is the most employed anode material in commercial LIBs because of its good structural stability and electrical conductivity

Despite its advantages, graphite presents several limitations, including a relatively low theoretical specific capacity (~372 mAh g^−1^) [17,18], leading to low energy density [19,20], as well as risks of thermal runaway under extreme mechanical, electrical, or thermal conditions [21,22,23]. Moreover, graphite anodes can suffer from safety issues associated with lithium dendrite formation [11]. During intercalation, graphite forms LiC_6_, in which one lithium atom is inserted between each carbon layer, thus constraining its storage capacity.

In the search of alternative materials, nanostructured metal oxides have emerged as promising candidates for LIB anodes [4,6,8,24,25,26]. Materials such as TiO_2_, NiO, CuO, Cu_2_O, Fe_2_O_3_, SnO_2_, WO_3_ and Co_3_O_4_ are being extensively investigated due to their higher capacity, structural stability, suppression of metallic lithium formation, and enhanced ionic and electronic transport properties largely attributed to their nanostructured morphology [4,6,8,24,25,26]. These oxides can react with lithium ions through two primary mechanisms: (1) insertion/intercalation reactions, where Li^+^ ions are reversibly accommodated within the oxide structure without significant phase transformations, and (2) conversion reactions, in which Li^+^ ions react with the metal oxide to form Li_2_O [6,24]. Depending on the dominant reaction pathway, as well as the morphology and crystallinity of the electrode material, different electrochemical behaviors and performance levels can be achieved.

Among the metal oxide candidates, nanostructured tungsten trioxide (WO_3_) plays a crucial role in the development of advanced LIB anodes, offering a promising alternative to graphite. WO_3_ nanostructures enhance battery performance by increasing surface area, improving charge/discharge rates, and thereby enhancing the overall energy efficiency of the device [27]. Additionally, WO_3_ exhibits high stability across a wide range of chemical environments, particularly under acidic conditions. During electrochemical cycling, the WO_3_ structure allows for the insertion of up to six Li^+^ ions per W^6+^ cation, highlighting its potential as a high-performance anode material for next-generation lithium-ion batteries [28].

In the present work, WO_3_ nanostructures were fabricated using electrochemical anodization in an acidic medium, with the aim of evaluating their potential use as anodes for lithium-ion batteries. The anodization process was carried out in the presence of three carboxylic acids: citric, tartaric, and L-aspartic acid, which acted as complexing agents for tungsten. The resulting samples were subjected to physicochemical (FESEM, XRD and TEM) and electrochemical characterization (EIS and ECSA), the influence of these complexing agents on the formation mechanisms was also investigated. Finally, the electrochemical performance, stability, and efficiency of the resulting WO_3_ nanostructures were evaluated in lithium-ion battery operation tests to determine which material exhibits the most favorable behavior.

## 2. Materials and Methods

### 2.1. Fabrication of WO_3_ Nanostructures

WO_3_ nanostructures were prepared by electrochemical anodization of tungsten in acidic electrolytes containing 1.5 M methanesulfonic acid, that is a good solvent for metallic salts and a strong acid, necessary for dissolution–precipitation mechanisms involved in WO_3_ nanostructures formation (pH below 1). It is less toxic than other strong acids. Furthermore, it is less harmful compared to other acids and, under normal conditions, does not generate hazardous volatile chemicals. The electrolyte also contained 0.1 M of various carboxylic acids (L-aspartic acid, citric acid, and tartaric acid). Methanesulfonic acid served as the primary electrolyte, whereas the carboxylic acids acted as complex agents for tungsten, influencing the growth mechanisms, morphology, and physicochemical properties of the resulting nanostructures. The anodization process was carried out at 50 °C under a constant applied potential of 20 V (using a power supply EA-PS 2384-05 B, Elektro-Automatik GmbH & Co. KG, Viersen, Germany) for 4 h, using a tungsten foil (0.5 cm^2^ exposed area) as the anode and a platinum foil as the cathode. The experiments were carried out using controlled hydrodynamic conditions employing a Rotating Disk Electrode (RDE, OrigaLys ElectroChem SAS, Lyon, France) setup operating at a stable rotation speed of 750 rpm to enhance the homogeneity of nanostructure formation. Following anodization, all samples were thermally treated in air at 600 °C (10 °C/min) for 4 h to promote dehydration and crystallization, leading to the formation of well-defined crystalline WO_3_ structures.

### 2.2. Physicochemical Characterization

WO_3_ nanostructures were morphologically and dimensionally examined by Field Emission Scanning Electron Microscopy (FESEM) using a Hitachi S-4800 instrument operated (Hitachi, Tokyo, Japan) at an accelerating voltage of 5 kV. The dimensional measurements were performed with the FESEM software (Digital Micrograph 3) imagen analysis and porosity was determined using ImageJ software (version 1.58 g). High-Resolution Transmission Electron Microscopy (HRTEM) was employed to further investigate the structural and morphological characteristics of the samples. The analyses were using a FEI Field Emission Gun (FEG) TECNAI G2 F20 S-TWIN microscope (FEI, Hillsboro, OR, USA) working at 200 kV. Before the TEM analysis, the samples were ultrasonically dispersed in ethanol (pure) for 5 min, and a drop of the resulting suspension was placed onto a holey carbon film supported on a copper grid and led to dry at room temperature. Crystallinity and the crystalline phase composition of the WO_3_ nanostructures were evaluated by X-ray diffraction (XRD) using a Bruker D8 ADVANCE A25 diffractometer (Bruker, Billerica, MA, USA) fitted up with a monochromator and CuKα radiation (λ = 0.15406 nm).

### 2.3. Electrochemical Characterization

Electrochemical measurements were carried out in a conventional three-electrode cell connected to a PalmSens4 potentiostat. A silver/silver chloride electrode (Ag/AgCl, 3 M KCl) served as the reference electrode, a platinum foil as the counter electrode, and the WO_3_ nanostructures (0.5 cm^2^ exposed area) as the working electrodes. All experiments were conducted at room temperature in 0.1 M H_2_SO_4_ aqueous solution (commonly used for these analyses due to its high conductivity and its acidic character, a medium in which WO_3_ is stable). Electrochemical impedance spectroscopy (EIS) measurements were performed at a fixed DC potential of 1 V with a sinusoidal perturbation of 0.01 V amplitude over a frequency range from 100 kHz to 10 mHz. The electrochemically active surface area (ECSA) was determined by cyclic voltammetry (CV). The open-circuit potential (OCP) of each sample was first established, and CV scans were then performed within a potential window of ±100 mV at scan rates of 10, 20, 50, 100, and 200 mV s^−1^. The ECSA values were calculated from the capacitive current densities recorded with varying scan rates, as detailed in Section 3.

### 2.4. Application as Anodes in Lithium-Ion Batteries

For evaluation as negative electrodes (anodes) in lithium-ion batteries, the WO_3_ nanostructures (theoretical specific capacity ~694 mAh g^−1^) were assembled into Swagelok-type cells. The WO_3_ nanostructure served as the working electrodes, while lithium metal foils were used as both counter and reference electrodes. Cell assembly was carried out in an argon-filled glovebox using 0.5 mL of 1 M LiPF6 in a 1:1:1 (*v*/*v*/*v*) mixture of ethylene carbonate, dimethyl carbonate, and diethyl carbonate (Sigma-Aldrich, St. Louis, MO, USA) as the electrolyte, and a glass microfiber separator (Whatman, Maidstone, UK). The assembled cells were tested using a Biologic BCS-815 battery cycler (BioLogic, Seyssinet-Pariset, France), at room temperature. Galvanostatic charge–discharge profiles were recorded at different current rates (C/20, C/10, C/5, C/2, and 1 C, followed by a return to C/20). The current corresponding to 1 C was ±1.318 mA (mass loading 3.8 mg/cm^2^), given that the mass of WO_3_ nanostructures obtained was comparable across the different carboxylic acid systems. All cycling experiments were conducted within a potential window of 0.0–3.5 V versus Li/Li^+^.

## 3. Results and Discussion

### 3.1. On the Synthesis of the Nanostructures: Anodization Curves Profile

**Figure 1:**
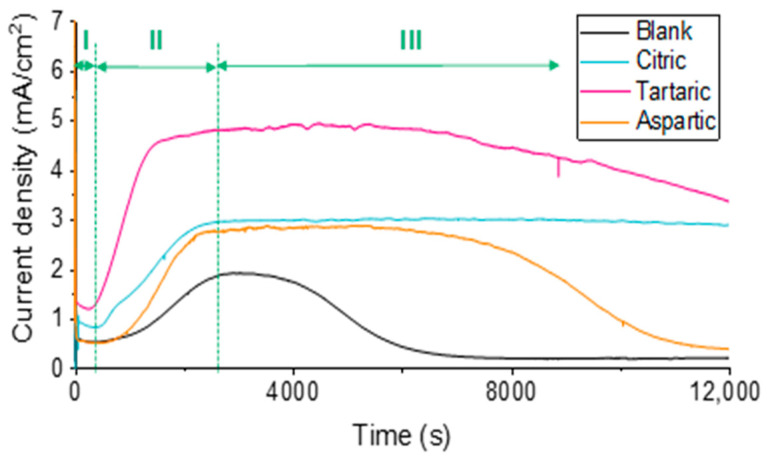
Anodization curves (current density vs. time) for the samples anodized in absence (blank) and in the presence of 0.1 M carboxylic acids. The different stages of the anodization process are indicated as I, II, and III.

Current density–time curves corresponding to the different WO_3_ nanostructures fabricated under various anodization conditions are shown in Figure 1. For comparison, a reference curve (blank sample) is also included, obtained in the absence of carboxylic acids (i.e., by anodization in 1.5 M methanesulfonic acid only). This reference serves to evaluate the influence of the complexing agents employed. All samples exhibit the typical behavior of tungsten anodization in electrolytes containing complexing species, which proceeds through a dissolution–precipitation mechanism [28,29,30]. Three main formation stages can be clearly distinguished. Figure S1 shows a synthesis scheme of WO_3_ nanostructures.

During Stage I, a sharp decrease in current density is observed immediately after applying the external potential (20 V), corresponding to the growth of a compact WO_3_ layer over the tungsten surface. Subsequently, this layer undergoes partial dissolution due to the attack of H^+^ ions and, more specifically, the carboxylic acids present in the electrolyte. This marks the beginning of Stage II, during which the current density increases because of oxide dissolution and the formation of soluble tungsten complexes. The extent of this increase depends on the complexing ability of each acid.

When anodized in the absence of carboxylic acids (blank sample), the current density rise is moderate, whereas in the presence of complexing acids, a more pronounced increase is observed, particularly with tartaric acid, which leads to the highest current density values.

Stage III is characterized by a gradual decrease in current density, corresponding to the precipitation of the soluble tungsten species formed during Stage II onto the electrode surface as hydrated WO_3_ nanostructures (tungstic acid). In the blank sample, this drop occurs earlier and more abruptly, with the current density approaching zero. This behavior suggests that the resulting oxide layer is denser and less porous than those obtained in the presence of carboxylic acids, confirming that H^+^ ions alone are not efficient complexing agents for tungsten; thus, supersaturation and precipitation occur rapidly.

Consequently, WO_3_ nanostructures grown in the blank electrolyte are expected to be larger and less porous. In contrast, the sample anodized in L-aspartic acid shows a somewhat faster current drop near the end of the process (after 4 h), suggesting the formation of a relatively compact WO_3_ layer with larger nanostructures. On the other hand, when citric acid or tartaric acid are used, the current density decreases more slowly and maintains higher values at the end of anodization, indicating the formation of more porous oxide layers capable of sustaining current flow.

Therefore, WO_3_ nanostructures synthesized in citric and, especially, tartaric acids are expected to exhibit smaller particle sizes and higher porosity, due to the greater stability of tungsten complexes in solution.

This behavior can be rationalized by taking into account the molecular structures of the carboxylic acids employed (Figure S2). In the case of citric and tartaric acids, both contain carboxyl groups whose oxygen atoms can act as bidentate ligands, providing strong metal–ligand coordination. Although structurally similar, anodization in tartaric acid results in higher current densities, suggesting a stronger complexing behavior toward tungsten. In contrast, L-aspartic acid, despite containing two carboxylic acid groups and one amino group, which in principle could enhance its complexation strength exhibits weaker complexing ability under acidic conditions. This agrees with the lower current densities observed during anodization in the presence of this acid.

### 3.2. Physicochemical Characterization of the Nanostructures: Morphological Analysis and Structural Characterization

**Figure 2:**
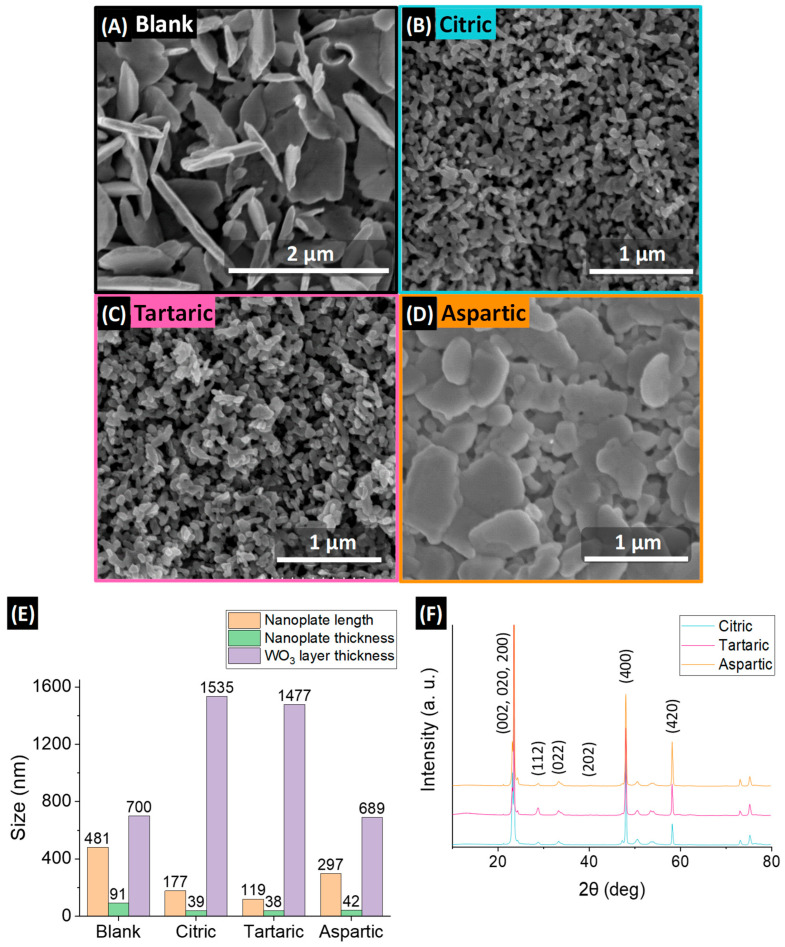
FESEM images of the WO_3_ nanostructures anodized in absence (**A**) and in the presence of 0.1 M carboxylic acids: citric (**B**), tartaric (**C**) and L-aspartic (**D**). (**E**) Comparison of the dimension and main thickness of the WO_3_ nanostructures synthesized by electrochemical anodization in absence and in the presence of 0.1 M carboxylic acids. (**F**) XRD spectra of the WO_3_ nanostructures fabricated in the presence of 0.1 M carboxylic acids (JCPDS: 43–1035).

The morphology and size of the anodized WO_3_ nanostructures were examined by field-emission scanning electron microscopy. Representative images are shown in Figure 2A–D.

For the blank sample (Figure 2A), plate-like nanostructures were observed uniformly distributed across the electrode surface, with an average length of 0.58 ± 0.08 μm. In the samples synthesized in the presence of citric acid (Figure 2B) and tartaric acid (Figure 2C), the morphology changed markedly, showing the formation of ordered arrays of nanoroads, which significantly increased surface porosity. The characteristic dimensions were 0.052 ± 0.006 μm (citric acid) and 0.062 ± 0.009 μm (tartaric acid), with estimated porosities of 26.6% and 36.4%, respectively. In contrast, anodization in L-aspartic acid (Figure 2D), again yielded plate-like structures, though with smaller dimensions (average size 0.30 ± 0.13 μm) and a porosity of 19.9%, lower than in the samples obtained with citric or tartaric acids. By way of illustration, an image of the WO_3_ nanostructure formed in the presence of citric acid but without thermal treatment is shown in Figure S3. This figure is presented just to confirm that thermal treatment did not affect the morphology of the samples.

The FESEM observations are coherent with the current density evolution recorded during anodization (Figure 1), confirming that higher porosity and smaller grain sizes are obtained when citric and tartaric acids are used during anodization. This trend was further verified by comparing the thickness of the WO_3_ nanostructured layers (Figure 2E): the thinnest nanoplates were obtained in tartaric acid, followed by citric acid, L-aspartic acid, and finally the blank sample.

The X-ray diffraction patterns of the WO_3_ samples synthesized with different carboxylic acids are presented in Figure 2F. All diffractograms exhibit peaks characteristic of monoclinic WO_3_ (JCPDS: 43–1035) [31,32], indicating that the crystalline phase is the same regardless of the acid used during synthesis. However, variations in peak intensity were observed: the strongest reflections correspond to the sample fabricated in L-aspartic acid, while the weakest were found in the sample fabricated with tartaric acid. The average crystallite size was estimated using the Scherrer equation, assuming a shape factor of k = 0.9 and an X-ray wavelength of 0.15406 nm (CuKα radiation). Only minor differences were observed between samples, with slightly larger crystallites in the L-aspartic acid system and the smallest ones in tartaric acid. The average crystallite sizes obtained were 39 nm for samples synthesized with the citric acid-containing electrolyte, 38 nm for those synthesized with tartaric acid, and 42 nm for the nanostructures obtained using L-aspartic acid.

**Figure 3:**
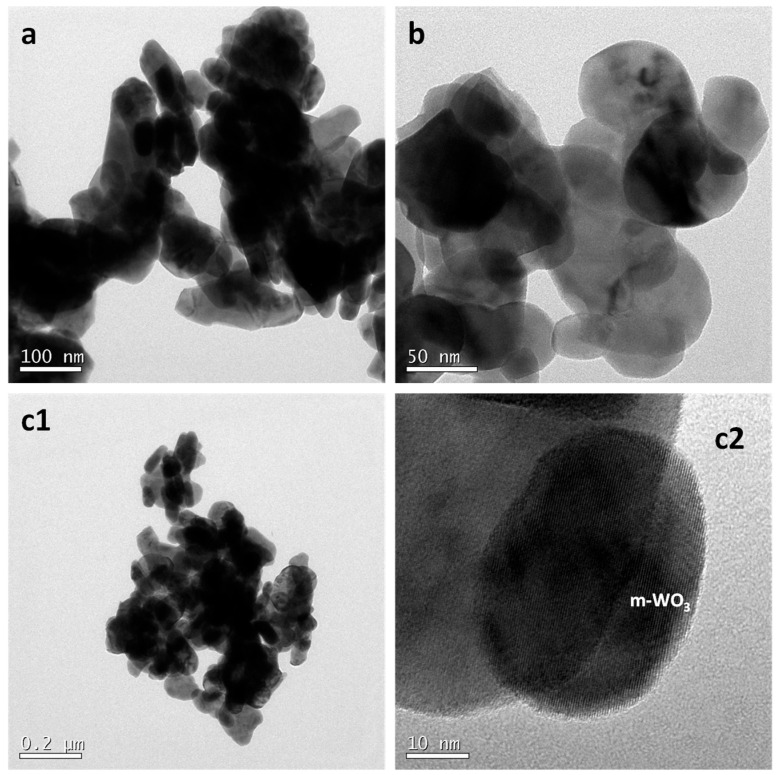
TEM images of the WO_3_ nanostructures synthesized in the presence of 0.1 M carboxylic acids: (**a**) L-aspartic, (**b**) citric and (**c1**,**c2**) tartaric.

Transmission electron microscopy (TEM) analyses supported the morphological trends observed by FESEM (Figure 3). The WO_3_ nanostructures synthesized in citric and tartaric acids exhibited similar morphologies, while those obtained in L-aspartic acid displayed noticeably larger and denser particles.

For the sample anodized in L-aspartic acid (Figure 3a), the structures appeared compact and elongated, with diameters in the range of 40–120 nm (length variable, depending on the scraping method) and low porosity. Additionally, small secondary nanoparticles (~10 nm) were observed decorating the main structures. When anodization was performed in citric acid (Figure 3b) or tartaric acid (Figure 3(c1)), the overall morphology remained similar, but the structures were significantly narrower, with diameters ranging from 20 to 50 nm. The nanostructures obtained in tartaric acid showed a more porous nature, which is expected to positively influence their electrochemical performance. High-resolution TEM images (Figure 3(c2)) revealed interplanar spacings of 2.64 Å and 3.67 Å, corresponding to the (202) and (200) Bragg reflections of monoclinic WO_3_ (JCPDS: 43-1035). No metallic tungsten was detected, confirming that the surface-scraping procedure was properly executed.

### 3.3. Electrochemical Characterization of the Nanostructures

**Figure 4:**
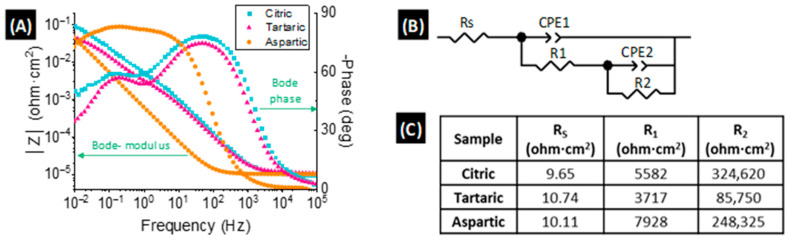
(**A**) Bode-phase and Bode-modulus plots for the EIS preformed to the WO_3_ nanostructures fabricated in the presence of 0.1 M carboxylic acids. (**B**) Equivalent circuit model used to fit the EIS values. (**C**) Resistance values for the electrolyte and WO_3_ layers of the samples, according to the equivalent circuit model. Experiments conducted at room temperature in 0.1 M H_2_SO_4_ aqueous solution.

EIS spectra of WO_3_ nanostructures prepared with electrolytes containing different carboxylic acids are presented in Figure 4A. The Nyquist plots (Figure S4) display two distinct semicircles, one at high frequencies and another at low frequencies, indicating the presence of two separate charge-transfer processes taking place at the electrode/electrolyte interface. These distinct behaviors are also clearly observable in the Bode phase plots (Figure 4A).

From the Bode modulus plots (Figure 4A), the characteristic resistances of the electrochemical system were extracted. The solution resistance (R_S_) was determined from the high-frequency intercept, whereas the total charge-transfer resistance (R_T_) of the WO_3_ nanostructures was obtained at low frequencies. Among the samples, the nanostructures anodized in citric acid exhibited the highest total resistance, while samples fabricated with tartaric and L-aspartic acids showed significantly lower values of R_T_.

To analyze EIS data more in depth, these data were fitted to an equivalent circuit model, consisting of two parallel resistor–constant phase element (R–CPE) branches (Figure 4B). The use of CPEs accounts for the non-ideality behavior of capacitors normally observed in real electrochemical systems [33,34]. In this circuit, R_S_ represents the solution resistance, the R_1_–CPE_1_ pair is related to the double-layer formed at the WO_3_/electrolyte interface, and the R_2_–CPE_2_ pair describes the resistive and capacitive properties of the WO_3_ compact film at the bottom of the nanostructure [35,36]. The extracted resistance values (Figure 4C), with fitting errors below 10^−3^, confirm the validity of the scratching model.

A lower total charge-transfer resistance (R_T_) indicates more efficient electrochemical processes during battery operation, leading to improved electronic conductivity and reduced material degradation. Specifically, R_1_, corresponding to the nanostructured WO_3_ layer, reflects the intrinsic charge-transfer kinetics within the nanostructure. Lower R_1_ values therefore imply faster interfacial reaction kinetics and enhanced electrochemical stability [37,38]. The irreversible loss of capacity, normally associated with the formation of the solid–electrolyte interphase (SEI), can also influence the resistance to total charge transfer, which would significantly influence the electrochemical processes of charge and discharge. With this premise, the R_1_ value offered by the material must be as low as possible, in order to counteract the additional resistance that the SEI will offer once formed [37,38,39,40].

Accordingly, the nanostructures synthesized in the presence of tartaric acid exhibited the lowest R_1_ values, followed by those prepared with L-aspartic acid, and finally those with citric acid, suggesting superior charge-transfer efficiency and structural robustness for the former.

**Figure 5:**
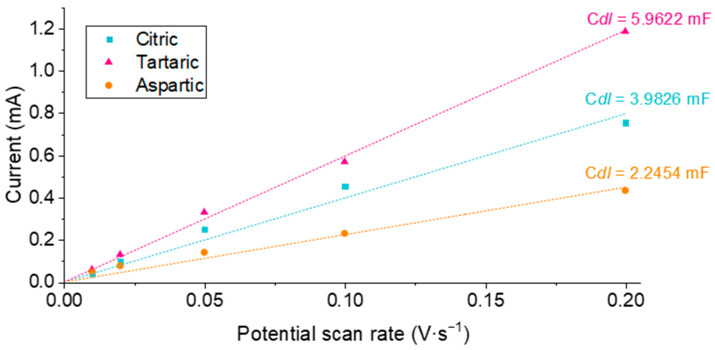
Anodic charging currents of WO_3_ nanostructures fabricated by electrochemical anodization in the presence of 0.1 M of different carboxylic acids. Experiments conducted at room temperature in 0.1 M H_2_SO_4_ aqueous solution.

The determination of the electrochemically active surface area has also been undertaken, since it is a paramount factor that can give insights into the behavior of the battery. Figure S5 shows the cyclic voltammograms recorded in the non-faradaic potential region at different scan rates for the various WO_3_ nanostructures. The double-layer capacitance (C_dl_) was determined from the slope of the plot of anodic current versus scan rate (Figure 5), following Equation [41], ECSA = C_dl_/C_s_. In this equation, C_s_ is the specific capacitance of the sample with an atomically smooth surface obtained under the same electrolyte conditions. This parameter is complicated to implement due to its own definition. Thus, the C_dl_ parameter serves as a semiquantitative indicator of the electrochemically active surface area of the electrode material.

Based on the results in Figure 5, the WO_3_ nanostructures synthesized in tartaric acid exhibited the highest C_dl_ values, and therefore the largest ECSA. Samples prepared in citric acid displayed intermediate C_dl_ values, while those anodized in L-aspartic acid showed the lowest, consistent with the previously discussed impedance behavior.

**Figure 6:**
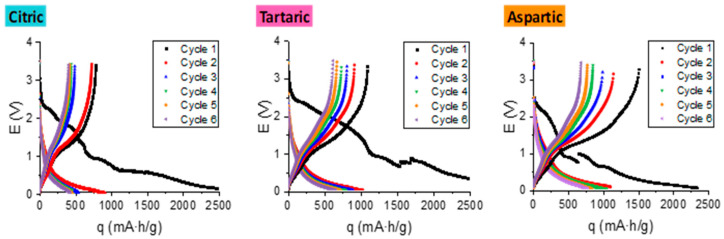
Charge/Discharge curves for LIBs using WO_3_ anodes synthesized in the presence of 0.1 M carboxylic acids.

Figure 6 presents the galvanostatic charge (Li^+^ insertion) and discharge (Li^+^ extraction from Li_6_WO_3_) curves recorded at a rate of C/20, following the global electrochemical reaction between Li ions and the WO_3_ nanostructures: ${WO}_{3}+6{Li}^{+}+6e^{-}\to W+{3Li}_{2}O$.

As previously discussed, each WO_3_ unit can theoretically accommodate up to six Li^+^ ions, enabling high electronic storage efficiency. Regardless of the acid employed in synthesis, all samples exhibited similar overall electrochemical profiles.

During the first charge–discharge cycle, a sharp decrease in specific capacity is observed, corresponding to the SEI formation between the electrode and electrolyte [42,43,44]. Remarkably, all WO_3_ anodes demonstrated specific capacities exceeding that of commercial graphite anodes (372 mAh g^−1^) [17,18].

When examining the charge and discharge curves (all measured at C/20), significant differences can be observed among the samples. In the charge curves obtained using the sample synthesized in the presence of L-aspartic acid, a pronounced separation between cycles can be seen, indicating a much greater capacity loss at the same cycling rate. This irreversible capacity loss, due to different transformation processes between the WO_3_ lattice and the Li^+^ ions [45,46,47,48,49], affects the overall battery performance (leading to reduced efficiency). In contrast, the curves obtained for the other samples (citric and tartaric acid) do not exhibit significant separation, which becomes almost negligible for the samples anodized with citric acid during the last three charge cycles.

The samples anodized with citric acid show a noticeable jump between the second and third cycles, which may indicate that the electrochemical processes occurring during this interval are largely irreversible. Unlike the samples anodized in the presence of 0.1 M L-aspartic acid, the samples anodized with citric acid regain a certain degree of stability after this process, and no further capacity losses of this nature are observed throughout the subsequent cycling.

Finally, the samples anodized with tartaric acid also show some degree of separation between their charge curves, although it is not significant. This suggests an irreversible capacity loss during the initial charge cycles, which tends to diminish as cycling proceeds. These samples exhibit greater long-term stability than those anodized with L-aspartic acid, but lower stability than the citric acid-anodized ones. Regarding the discharge curves, the separation is generally negligible across most cycles, regardless of the anode sample used.

As it can be seen from the charge/discharge curves, the morphological and structural differences induced by the choice of acid significantly affected the electrochemical behavior. WO_3_ nanostructures anodized in tartaric acid achieved the highest specific capacities, 3122.98 mAh g^−1^ (charge) and 1089.70 mAh g^−1^ (discharge) corresponding to an Initial Coulombic Efficiency (ICE) of 35%, followed by those obtained with citric acid (2778.95 mAh g^−1^ and 835.23 mAh g^−1^), with an ICE of 30% and L-aspartic acid (2777.79 mAh g^−1^ and 781.58 mAh g^−1^) with an ICE of 28%.

SEI formation is not the only cause of the specific capacity drop in the first charge/discharge cycle, but there are other irreversible processes that are also reflected in this loss. These include the decomposition of the electrolyte at low potentials, the permanent insertion of lithium into the WO_3_ lattice produced by irreversible phase transformations, and the reduction of W^6+^ to W, responsible for the destructive or deformation processes of the WO_3_ crystalline structure [28]. These processes should be studied and optimized in the future to reduce capacity loss.

These results are consistent with the EIS analysis, as the specific capacity strongly correlates with total charge-transfer resistance: lower *R*_1_ values enable faster and more efficient redox reactions involving Li^+^ ions. Additionally, the larger ECSA of the tartaric acid-derived samples further enhances their electrochemical response, supporting their superior performance.

**Figure 7:**
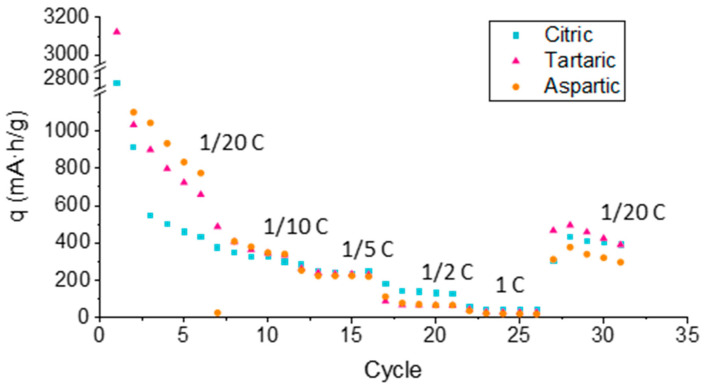
Specific capacity (charge) vs. number of cycles of the different WO_3_ samples anodized in the presence of 0.1 M carboxylic acids.

A comprehensive assessment of the cycling stability and rate capability is necessary to fully characterize the performance of the tungsten oxide nanostructures. Figure 7 illustrates the variation in maximum specific capacity as a function of cycle number at different C-rates. As expected, higher cycling rates result in a reduction in capacity due to kinetic limitations. However, upon returning to slower cycling rates, capacity recovery was observed, indicating good structural reversibility. The degree of recovery provides insight into the stability of the nanostructures: a desirable material should maintain its structural integrity under fast cycling and partially recover its capacity afterward. Among the samples, the WO_3_ nanostructures synthesized in citric acid exhibited the best recovery, closely followed by those prepared in tartaric acid and L-aspartic acid.

While the recovery behavior of the citric and tartaric acid samples was comparable, the tartaric acid-derived nanostructures consistently delivered higher specific capacities throughout the cycling process, as also reflected in the charge–discharge curves. Thus, although the citric acid-based samples showed slightly better recovery, the tartaric acid-derived WO_3_ nanostructures demonstrate the best overall electrochemical performance, combining high capacity, efficient charge transfer, and good structural stability.

Results for the blank sample have been extracted from a previous work [28] in order to compare them with the results obtained in this study. Blank sample exhibited a specific capacity of 255 mAh g^−1^ in charge and 248 mAh g^−1^ in discharge (measured at 1/10 C after 5 complete cycles). If compared with specific capacities at the same charge/discharge rate and for the same amount of cycles (between 300–350 mAh g^−1^), is can be seen that nanostructures anodized using carboxylic acids presented better properties for Li^+^ insertion/extraction.

## 4. Conclusions

In the present work, WO_3_ nanostructures were obtained by electrochemical anodization in acidic media into electrolytes containing different carboxylic acids: citric, L-aspartic and tartaric acid. The morphology and crystallinity, analyzed by FESEM and TEM imaging together with X-ray diffraction patterns, confirmed the exclusive formation of monoclinic WO_3_. Furthermore, electrochemical characterization of the samples was performed.

EIS revealed that the samples anodized in the presence of tartaric acid exhibited the lowest overall charge-transfer resistance, as well as the smallest charge-transfer related to the nanostructured WO_3_ layer. These samples also displayed the highest electrochemically active surface area, indicating more efficient electrochemical processes, enhanced conductivity, and improved stability. Consequently, these samples represent promising anode candidates, delivering an initial specific capacity of 3122.98 mAh g^−1^ during charge and 1089.70 mAh g^−1^ during discharge, values exceeding those obtained for the other samples and surpassing the initial capacity of commercial graphite anodes. Moreover, samples prepared in the presence of tartaric acid demonstrated remarkable capacity recovery after cycling at higher rates (highly demanding conditions), reaching values of capacity that, again, are above those achievable with commercial graphite.

## Data Availability

The data presented in this study are available on request from the corresponding author due to privacy restrictions.

## Supplementary Materials

The following supporting information can be downloaded at: https://www.mdpi.com/article/10.3390/ma18245602/s1. Figure S1: Synthesis scheme of the WO_3_ nanostructures by electrochemical anodization in presence of 0.1 M carboxylic acids. Figure S2: Molecules of carboxylic acids used in the electrochemical anodization of tungsten. Figure S3: FESEM image of the sample anodized in the presence of citric acid before the thermal treatment at 600 °C for 4 h. Figure S4: Nyquist diagram of WO_3_ nanostructures synthesized by electrochemical anodization with different carboxylic acids. Figure S5: Cyclic voltammetry measured in a non-Fradaic region at different potential scan rates of WO_3_ nanostructures synthesized by electrochemical anodization with different carboxylic acids.
