# Supplementary material for: Engineering WO3 Nanostructures via Carboxylic Acid Anodization for Advanced Lithium-Ion Battery Anodes
Source: Materials (Basel). 2025 Dec 13;18(24):5602. doi: 10.3390/ma18245602 (PMC12735234; doi:10.3390/ma18245602)
Supplement: Supplementary file 1 [file materials-18-05602-s001.zip › materials-4029655-supplementary.pdf]

Supporting information

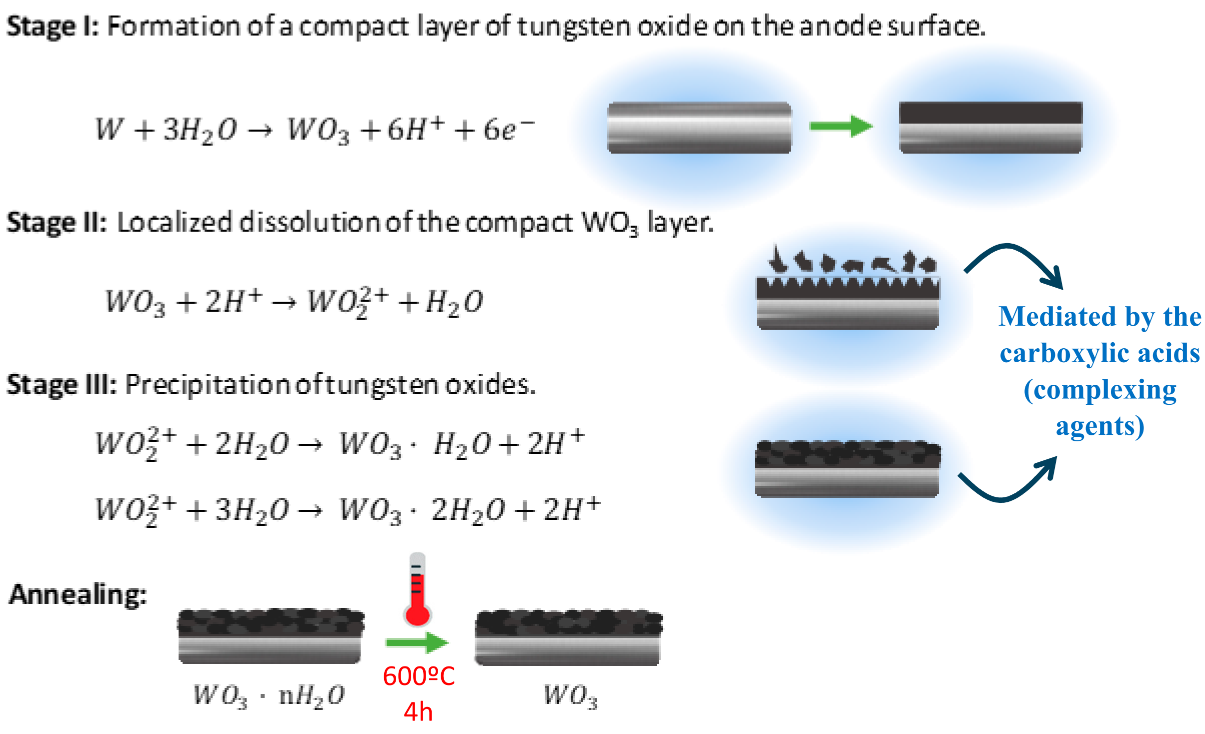

Figure S1: Synthesis scheme of the WO_3 nanostructures by electrochemical anodization in presence of 0.1 M carboxylic acids.

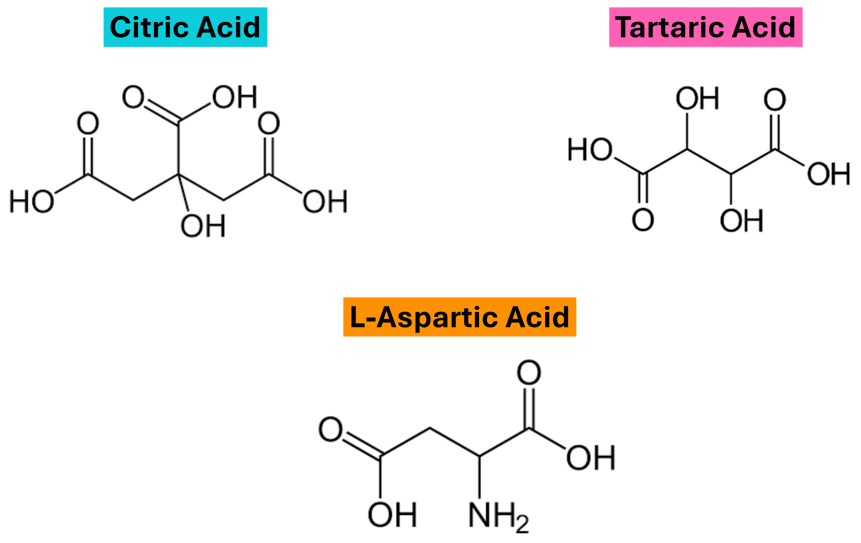

Figure S2: Molecules of carboxylic acids used in the electrochemical anodization of tungsten.

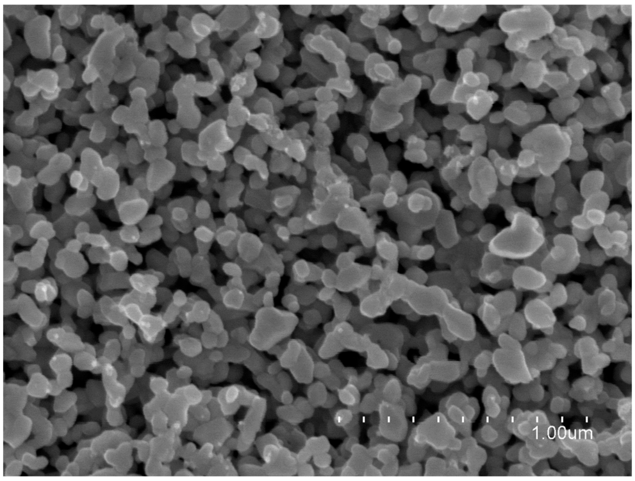

Figure S3: FESEM image of the sample anodized in the presence of citric acid before the thermal treatment at 600ºC for 4h.

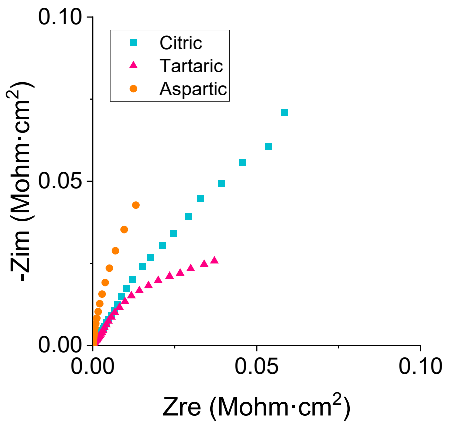

Figure S4: Nyquist diagram of WO_3 nanostructures synthesized by electrochemical anodization with different carboxylic acids.

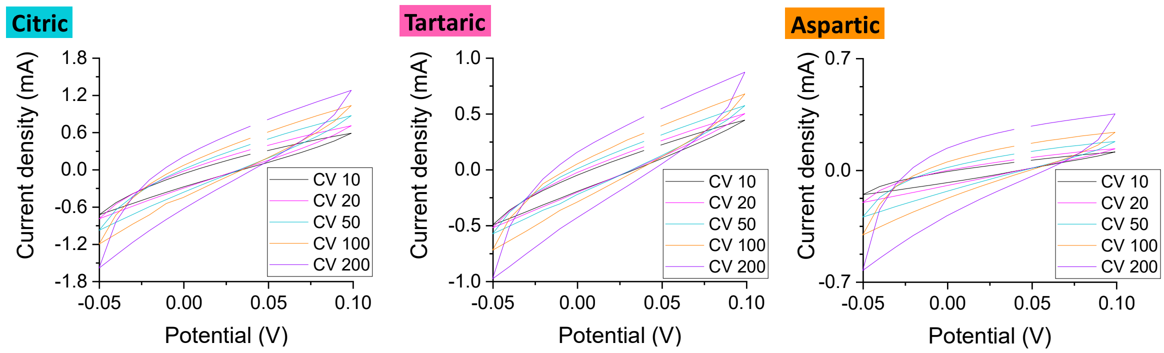

Figure S5: Cyclic voltammetry measured in a non-Fradaic region at different potential scan rates of WO_3 nanostructures synthesized by electrochemical anodization with different carboxylic acids.
